# What's in a virus? Folk understandings of hepatitis C infection and infectiousness among injecting drug users in Kings Cross, Sydney

**DOI:** 10.1186/1475-9276-4-5

**Published:** 2005-03-23

**Authors:** Erica Southgate, Anne Maree Weatherall, Carolyn Day, Kate A Dolan

**Affiliations:** 1 Centre for Clinical Epidemiology and Biostatistics, University of Newcastle, Sydney, Australia; 2 National Drug and Alcohol Research Centre, University of New South Wales, Sydney, Australia

## Abstract

**Background:**

To explore folk understandings of blood borne virus infection and infectiousness among injecting drug users in Kings Cross, Sydney.

**Methods:**

Observational fieldwork was conducted in Kings Cross over a four month period. In-depth interviews with 24 current injectors and 4 key informants recruited from King Cross were undertaken.

**Results:**

Hepatitis C (HCV) generated different meanings from HIV. HIV was considered "the dreaded" and generated fear of infection and dire disease progression. Whereas HCV was considered non-desirable but less threatening than HIV. The risks of transmitting HCV through sharing injecting paraphernalia was poorly understood. Some believed HCV infection was linked to poor hygiene and dirty water. Jaundice was mistakenly thought to indicate HCV infection and was used to gauge infectiousness. Many were confused about their current hepatitis C serostatus. Some participants thought they had a "dormant antibody" or that they had a "mild case" of infection. Participants were unsure what this meant for their own health or for their potential to infect others.

**Conclusion:**

Participants displayed confusion about transmission risks for hepatitis C, conflating blood awareness and hygiene health promotion messages. Participants' reliance on the symptom of jaundice to gauge serostatus places them at risk of transmitting and contracting HCV. Participants were confused about what a positive HCV diagnosis meant for their own health and their ability to infect others. Education is needed to debunk misconceptions about jaundice and clarify medical terms such as 'antibody' at the time of diagnosis. Further clarification of messages about injecting hygiene and blood awareness are also required.

## Introduction

An estimated 210,000 people are living with the hepatitis C virus (HCV) in Australia [1]. HCV is transmitted mainly through the shared use of injecting equipment by injecting drug users (IDUs) [2,3]. HCV is the most prevalent blood-borne virus infection among Australian IDUs, varying from almost 90% to just under 50%, depending on the injecting population sampled [4-7]. HCV infection is a major public health concern. High prevalence combined with high rates of new infection, continue to result in a large number of chronically ill people, a percentage of whom will develop cirrhosis of the liver and hepatocellular carcinoma [8,9]. Moreover, the potential cumulative health care costs of HCV infection over the next 60 years will be approximately $4 billion [10].

Epidemiological and quantitative behavioural research on hepatitis C has concentrated on documenting risk behaviour and knowledge of hepatitis C transmission risks among injecting drug users [2,3,7,11,12]. Qualitative research has sought to contextualise risk behaviour, enhancing the findings from quantitative studies [13-15]. Few studies have sought to uncover the ways IDUs themselves make sense of the medical terms and clinical markers associated with hepatitis C [16]. This includes the way injectors differentiate between blood-borne viruses; folk interpretations of medical terms and the symptoms of disease; and the links between these lay understandings and risky injecting. This article explores, using qualitative data, folk understandings of the hepatitis C virus among a group of IDUs who live in or frequently visit Kings Cross, Sydney.

The research utilised a socio-cultural approach to documenting and interpreting risky injecting practice [17-19]. It aimed to provide a window into the world of marginalised injecting drug users [20]. It sought not only to measure injectors' knowledge of hepatitis C against an expert standard of "right or wrong" fact, but also to reveal injectors' understandings of the virus as a real and threatening entity in their everyday lives. The significance of the approach lies in its ability to reveal interfaces between lay and expert knowledge: injectors take up the clinical language of medicine and health promotion and actively use it to make sense of living with HCV and to assess the likelihood of infecting others. The research therefore informs prevention efforts and education initiatives devised to inform people of the implications of their HCV status.

## Method

### Site selection

The study took place in Kings Cross, Sydney, between July 2001 and February 2002. Kings Cross is home to a dynamic, open-air drug market and a "red light" district. The drug market offers opportunities for small and large scale drug dealing in heroin, and more recently, cocaine. A range of other drugs such as ecstasy, amphetamine and methamphetamine are also available. Kings Cross provides an interesting site for the study of injecting networks for several reasons. The dynamic, open-air nature of the drug market facilitates aspects of qualitative field work including observation of public injecting and a heightened awareness of issues among health workers (which in turn translates into research questions). Kings Cross is the home of numerous health and welfare services, many of which target IDUs. No other area in Australia has so many services targeting IDUs, coupled with the long history of health initiatives aimed at preventing blood-borne virus infection (BBVI) transmission since the arrival of HIV in the mid 1980s. This study posed questions regarding the continuation of risk practice in an environment where free, sterile injecting equipment is readily available and education about BBVI transmission is constantly being delivered by health care workers.

### Data sources and sample

Three methods were used for data collection. Firstly, on-going contact with four key informants, including a local "guide" who was familiar with multiple injecting networks, provided continuous information on the state of the Kings Cross drug market and its impact on risk practice. Key informants were chosen because they were considered opinion leaders in their injecting network [21]. To facilitate field work, the research team asked service providers to nominate a guide [22]. The guide escorted the researcher through a variety of injecting locations, introduced the researcher to local identities and assisted in the selection of participants for in-depth interviews. The guide and other key informants helped to verify the accuracy of information as it emerged during the course of the study. Conversations with key informants were recorded in field note form.

Secondly, observational fieldwork was conducted between November 2001-February 2002. Approximately 300 hours were spent in the field. Fieldwork involved mapping the range of public, semi-public and private injecting locations in Kings Cross. Mapping of physical locations was complemented by the identification of networks of injecting drug users who frequented these sites. Limited observations of in-situ injecting episodes also occurred. Observations were recorded in field note form.

Thirdly, in-depth semi-structured interviews were conducted with IDUs living in or visiting the Kings Cross area. An interview schedule guided the process. The schedule was developed collaboratively by the investigators and key informants. The schedule comprised questions on drug use history, current injecting practice, BBVI knowledge and risk scenarios. Over three quarters of interview participants were recruited through the guide or by chain referral (snowballing). Interviews were conducted in a rented room at a welfare agency, in cafes and in public locations. Interviews lasted between thirty and ninety minutes.

Ethics approval was obtained from the University of New South Wales Human Research Ethics Committee. Informed written consent was obtained for interviews. Participants received up to $30 (Aus) as reimbursement for their time. The guide was employed at casual research officer rates.

In all, twenty four interviews were conducted, fourteen with men and ten with women. Ages ranged from 19 to 47 with most interviewees in their thirties. One participant was HIV positive. It was difficult to assess, on self-report, the HCV status of participants as there was confusion regarding current status, although most believed themselves to have been infected at some time. Participants primarily injected heroin, with most injecting cocaine when it was available. Few participants had stable housing, however most were homeless or itinerant, their lives characterised by social marginalisation, poverty and extreme poor health.

### Analysis

In line with Smith [23] and Glaser and Strauss [24], field notes and interview data were analysed in an on-going manner. Interviews and field notes were coded for key words, themes, issues and events. These were compared, contrasted and synthesised to create a system of thematic classification. A number of processes were used to assess the validity of the analysis. First, the research team read all the transcripts and field notes and analysed these data for contexts for BBVIs transmission as identified in the literature; common concerns of and discourses used by participants; similarities and disparities in network membership and environmental location for injecting; and collective and individual patterns of social action that make up everyday life. Data were re-read for disconfirming evidence. A process of theoretical validity was undertaken to ensure that the units of classification (themes, issues, concepts) were sensitive to the accounts supplied by participants and to scholarly literature [25]. Theoretical validity extended to evaluating the internal logic of and relationships between units of analysis [26]. Validity involved the use of a collective process of checking, questioning and theorising [27].

## Results

### Mixing messages: Folk understandings of 'Blood Awareness' and Hygiene Health Promotion messages

For most participants, HCV generated significantly different meanings from HIV. HIV was colloquially referred to as "the dreaded", a term encapsulating fear of infection and dire disease progression. HIV elicited strong reactions, in particular the desire to prevent infection by avoiding certain types of injectors considered to be at high risk such as gay men, or those individuals 'known' to be infected with the virus. In contrast to HIV, the meanings attached to the hepatitis C virus were fairly diverse. While hepatitis C infection was not considered desirable, participants viewed HCV as less damaging to health and quality of life than HIV. However, there was little evidence of a forlorn fatalism in regard to HCV infection: it was not considered attractive or inevitable. Nor was it viewed as a 'badge' signifying the position of a 'real user' contrary to previous research [28]. Pragmatism rather than fatalism predominated: if participants could avoid sharing needles (needle-sharing being the focus of folk infection-control) then they would. In cases where circumstances were deemed to prevent safer use then risks would be taken.

The concentration on preventing needle-sharing reflected the success of earlier HIV prevention campaigns based on the 'new fit for every hit' message. Some participants had good knowledge of 'blood awareness' health promotion messages, that is of the risks associated with transmitting HCV via the use of blood contaminated injecting paraphernalia and through touching others with bloody fingers etc. About half of the participants were less cognisant of these means of transmission.

Indeed, a number of participants held the view that hepatitis C was transmitted "through dirt". In many cases participants were re-interpreting public health hygiene messages. These messages refer to the benefits of hand-washing and wiping surfaces after injecting to prevent the inadvertent spread of HCV via touch and environmental blood transmission. HCV transmission was associated as much with unhygienic practice and "dirty, desperate" people as it was with technical blood-to-blood transmission routes [29]. Some participants, like Bruce, provided explanations for contracting HCV which involved both hygiene and blood awareness messages:

*Well I'm hep C positive at the moment and have been for quite a long time and I think that came about when I wasn't using sterile injecting water. I was taking water out of the toilets and things like that and using that to inject and I think that's how I got hep C. [Interviewer: So it wasn't sharing needles with other people?] No. It could have been either that or finding somebody else's blood or something in one of the packets (containing a drug deal) or something and just continuing to have what was left of the packet or something like that that caused me to get it. Or it could have been just using a very, very old syringe where the blood had gone off or something. (Bruce, a 30 year old injector)*.

Bruce's account is indicative of the way participants associated HCV transmission with both unhygienic practice and scientifically known transmission routes. Bruce variously suggests he may have contracted HCV from toilet water; from blood left in an old deal packet (one assumes a small plastic bag); or from re-using a "very, very, old syringe". Bruce demonstrates good technical knowledge when he states that HCV can be transmitted through blood contamination in drug deal packets. He also exhibits a commonly held understanding that HCV transmission is related to unhygienic practice. In this case unhygienic practice involves the use of toilet water and blood that has "gone off" in a syringe of extreme age.

The hygiene factor is also apparent in some participants' misconception that it is possible to infect ones-self with HCV through the re-use of one's own needle. In this case, the hepatitis C virus is not viewed as an agent which is external to the self, that is, it is caught from other people. Rather hepatitis C infection is thought to result from the "unclean" practice of re-using one's own needle. This misconception is based on the belief that one's own blood, once lodged in a used syringe, is capable of generating HCV as it changes or "goes off". According to this folk understanding, the health threat is endogenous to the drug user's body rather than exogenous or external [30].

### Reading the jaundiced body

Understandably, many participants were confused about the differences between hepatitis A, hepatitis B, and hepatitis C. The most obvious sign of this was the description participants gave of their own and others HCV seroconversion illness. Numerous participants stated that they "knew" they had contracted hepatitis C when they became jaundiced. Unlike hepatitis A and hepatitis B, jaundice is rarely associated with the acute phase of hepatitis C infection [31]. The commonly held assumption that hepatitis C status can reliably be determined by symptomatic jaundice has implications for prevention. In this case, infection and infectiousness is associated with a symptom that is much more likely to occur in the acute phase of hepatitis A and hepatitis B infection, not HCV infection. Participants actively 'read' their own bodies and the bodies of others, for jaundice. Jaundice was considered a reliable sign on which to assess hepatitis C status and the subsequent risks in lending or borrowing injecting equipment. Given the reliance some IDUs place on jaundice as a marker of HCV infection and infectiousness means that many may only seek HCV testing if they experience jaundice. Also some may well be basing their assumed negative status on the fact that they have not experienced jaundice. Michelle, a 35 year old injector, explains this logic:

I mean, touch wood, I never got AIDS or anything like that, and how I was diagnosed I was staying in a half-way house, rehab type of thing...Once a week we'd do groups on women, health issues and things like that and this one week was about Hep C. And he (a doctor) said, 'Hands up the people that have got it' and everyone put their hand up except for me and I said, 'Well, I've not been tested...but I can't remember being yellow or anything like that."...He said 'You don't necessarily go yellow. Can you remember in the last five years having a really bad flu?'

### Everybody's antibodies: Folk understandings of clinical terms

Reading the interview data as a whole, there is the distinct impression that many IDUs, at least half, were perplexed about the implications of a hepatitis C diagnosis. Most, at least two thirds, state that they are "carriers" who have "cleared" the virus or that the virus is currently "dormant". When asked about their current HCV status, over half of the participants spoke of having "antibodies" but were unsure what this meant in terms of their prognosis or their ability to infect others. Approximately ten participants recounted the fear and distress they felt after receiving a positive diagnosis. The lack of adequate hepatitis C pre and post test counselling has been documented in other research [16,32]. The following interview revealed the confusion and fear that can accompany a positive diagnosis:

*I haven't got AIDS and I haven't got Hep B but what I was told was that I carry the antibodies for (Hep) C, which I said 'What's that mean?'. Apparently it's that I might have a mild case of it and don't have it any more...They still want to follow it up now at the clinic and have a biopsy- cut a piece of my liver, test my liver...I should have went back (to the clinic) but that scared me. (Diane, a 29 year old injector)*.

Some participants, like Diane, spoke of having a "mild" or "small" case of HCV. Other used the clinical term 'antibodies' to describe having a resistance to HCV, somewhat like acquiring antibodies after having chicken pox. For example, Ken, a 22 year old injector, stated: "*I've been tested for Hep C. Been cleared and then the doctor said you got antibodies against it and, then he said there's no sign of it in your system*." Another, Glen, a 30 year old who had shared needles in jail, thought he was "*one of those strange people that might be able to fight it (hep C)*", thus perpetually avoiding infection. Phoebe, describes how proximity to someone else's hepatitis B and hepatitis C antibodies provided protection for her:

*I had a test...and they couldn't tell how me if I was a carrier or whether I had hepatitis A years ago. I had a hep B test and they said 'You're a carrier', and I said, 'Hang on, I've never had hepatitis in my life, never in my life.' 'Well', they said, 'you have been close to someone who has antibodies and that's a good thing'. I had a hep C test and they could only tell me the same thing. (Phoebe, a 42 year old injector)*.

Only a few participants understood that although they had spontaneously "cleared" the HCV, they were still open to reinfection. Similarly, only a couple of participants demonstrated knowledge of superinfection, that is infection with multiple genotypes of HCV. Confusion around diagnostic explanations, such as 'antibodies present' and 'virus cleared', left participants vulnerable to hepatitis C infection. These terms gave a false impression that injectors were protected from reinfection and superinfection because of the presence of antibodies.

## Discussion

Marginalised IDUs experience a syndemic pattern of health concerns, that is, they are affected by a set of synergistic and mutually enhancing health and social problems [33]. Public health concerns about hepatitis C should be located within the syndemic nature of the health and welfare issues facing marginalised people, such as those who participated in this study. Within a syndemic context, "risk" has myriad meanings, including but not solely focused on, risks for the transmission of hepatitis C. While the majority of participants were concerned about contracting and transmitting hepatitis C, this concern was only one of many which permeated everyday life. Hepatitis C did not have the immediacy of "the dreaded" HIV. Infection with hepatitis C is common among IDUs' friends and acquaintances. In the minds of IDUs, hepatitis C infection offered an uncertain future, but unlike HIV, a future none-the-less.

Indeed, hepatitis C was really the uncertain virus. For many participants there was uncertainty about modes of transmission other than through the sharing of needles. Some participants blended blood awareness messages with hygiene ones. This blending of messages created the misconception that "dirty" behaviour and "dirty" people were the cause of HCV infection. Participants were perplexed about the differences between the various hepatiti and the symptoms of their acute phases. Confusion accompanied hepatitis C diagnosis, with participants actively reinterpreting the medical language of their diagnostic experience.

Lay people invariably read their bodies and the bodies of others for signs of infection and illness [29,33]. So too do they appropriate expert knowledge, including medical terms and clinical markers, making sense of these at individual and collective levels [34]. Sometimes medical terms and clinical markers are actively used in the assessment of risk practice [35]. Prevention efforts should continue to reiterate messages about the risks of sharing needles and other injecting paraphernalia.

However prevention needs to be expanded to include education about the hepatitis C virus itself. Clarification, in accessible language, about the differences between the hepatiti is required, in particular the fact that jaundice is not a usual symptom of hepatitis C acute infection. The IDUs in our study were using the symptom of jaundice as a marker of infection and as a sign that they were capable of infecting others. IDUs should be informed that jaundice is not a reliable sign for gauging either infection or infectiousness. Similarly they should be advised that they should not wait for signs of jaundice before being tested for HCV.

On the subject of HCV testing, the findings from this study support other research [16,32] which suggests improvements are needed in pre and post test counselling, and in the provision of long-term support and information mechanisms for people with HCV, particularly marginalised IDUs. Alleviating the fear surrounding the testing experience and a positive HCV diagnosis is vital if IDUs are to be encouraged to seek testing and treatment.

The participants in this study all frequented the Kings Cross area where the range of drug services is the best in Australia, yet these IDUs were still engaging in risk behaviour and were unsure about a number of issues around hepatitis C. This suggests that even more support is required for these people to change their behaviour. One approach in Amsterdam has been the provision of case managers who arrange housing, methadone treatment and access to an injecting room for each client.

Moreover, the complexity of hepatitis infection, that is, the possibility the virus can be spontaneously cleared and the prospect of reinfection and superinfection, requires explanation among this group. Professionally supported peer education might be an efficient means of 'spreading the word' about these matters [22,36]. Situating public health certainties about the hepatitis C virus within the context of marginalised injecting networks has proved a challenging task. Targeted and accessible education and health promotion messages are required to unravel the complexities of HCV and its implications for acute and chronic infection and infectiousness. On-going examination of folk understandings of medical terms and clinical markers is a must if prevention and support efforts are to be successful in controlling the HCV epidemic.

## References

[B1] Law M, Dore G, Bath N, Thompson S, Crofts N, Dolan K, Giles W, Gow P, Loveday S, Powell E, Spencer J, Wodak A (2003). Modelling hepatitis C virus incidence, prevalence and long-term sequelae in Australia, 2001. International Journal of Epidemiology.

[B2] MacDonald M, Crofts N (1996). Transmission of hepatitis C virus. Epidemiologic Reviews.

[B3] Crofts N, Jolley D, van Beek I, Wodak A (1997). Epidemiology of hepatitis C virus infection among injecting drug users in Australia. Journal of Epidemiology and Community Health.

[B4] Loxley W, Carruthers S, Bevan J (1995). In the Same Vein: First report of the Australian Study of HIV and Injecting Drug Use (ASHIDU).

[B5] Crofts N, Aitken C (1997). Incidence of bloodborne virus infection and risk behaviours in a cohort of injecting drug users in Victoria, 1990–1995. Medical Journal of Australia.

[B6] Selvey LA, Denton M, Plant AJ (1997). Incidence and prevalence of hepatitis C among clients of a Brisbane methadone clinic: factors influencing hepatitis C serostatus. Australian and New Zealand Journal of Public Health.

[B7] MacDonald M (2001). HIV and HCV Infection and Related Risk Behaviour Among Injecting Drug Users at Selected Needle and Syringe Programs.

[B8] Afdhal NH (2004). The nature history of hepatitis C. Seminars in Liver Disease.

[B9] Seeff LB (1997). Natural history of hepatitis C. Hepatology.

[B10] Brown K, Crofts N (1998). Health care costs of a continuing epidemic of hepatitis C virus infection among injecting drug users. Australian and New Zealand Journal of Public Health.

[B11] van Beek I, Dwyer R, Dore GJ, Luo K (1998). Infection with HIV and hepatitis C virus among injecting drug users in a prevention setting: retrospective cohort study. British Medical Journal.

[B12] Dwyer R, Fry C, Carruthers S, Bolleter A, Dolan K, Donald A, Byrne J, Loxley W (2002). ABRIDUS: The Australian blood-borne virus risk and injecting drug use study A study of hepatitis C risk practices and contexts in Melbourne, Perth and Sydney.

[B13] Maher L, Dixon D, Lynskey M, Hall W (1998). Running the Risks: heroin, health and harm in South West Sydney, Research Monograph 38.

[B14] Maher L, Dixon D, Taylor S (2002). Policing and public health: Law enforcement and harm minimization in a street-level drug market. Ethnographic research: A reader.

[B15] Southgate E, Hopwood M (1999). The drug use and gay men project issue papers.

[B16] Loxley W, Bolleter A, Carruthers S (2001). Talking about testing: opportunities for prevention in blood borne virus testing and vaccination with injectors.

[B17] Douglas M (1992). Risk and blame Essays in cultural theory.

[B18] Petersen A, Lupton D (1992). The new public health Health and self in the age of risk.

[B19] Lupton D (1999). Risk, Routledge.

[B20] Hendry J (1999). An introduction to social anthropology: Other people's worlds.

[B21] Watters J, Biernacki P (1989). Targeted sampling: Options for the study of hidden populations. Social Problems.

[B22] Power R, Jones S, Kearns G, Ward J, Perera J (1995). Drug user networks, coping strategies, and HIV prevention in the community. Journal of Drug Issues.

[B23] Smith L (1979). An evolving logic of participant observation, educational ethnography, and other case studies. Review of Research in Education.

[B24] Glaser B, Strauss A (1967). The Discovery of Grounded Theory: Strategies for qualitative research.

[B25] Glucksmann M, Maynard M, Purvis J (1994). The work of knowledge and the knowledge of women's work. Researching women's lives from a feminist perspective.

[B26] Maxwell J (1992). Understanding validity in qualitative research. Harvard Educational Review.

[B27] Kvale S (1996). Interviews: An introduction to qualitative research interviewing.

[B28] Southgate E, Kippax S, Owler K (2002). Social research needs analysis: Australian Intravenous League and Australian Hepatitis Council and member organisations.

[B29] Hughes R (1999). "Clean" and "dirty": Drug injectors perceptions of cleanliness and dirtiness in relation to HIV risk behaviour. Addiction Research.

[B30] Herzlich C (1973). Health and illness: A social psychological analysis.

[B31] Australian Society for HIV Medicine (2001). Hepatitis C: The facts, Sydney.

[B32] Gifford S, O'Brien M, Banwell C, Bammer G (2001). A Survey of women living with hepatitis C in Victoria and the ACT.

[B33] Singer M (1994). AIDS and the health crisis of the urban poor; The perspective of critical medical anthropology. Social Science and Medicine.

[B34] Turner B (1996). Medical power and social knowledge.

[B35] Rosengarten M, Race K, Kippax S (2000). Touch wood everything will be OK: Gay men's understandings of clinical markers in sexual practice.

[B36] Latkin C, Mandell W, Vlahov D, Knowlton A, Oziemkowska M, Celentano D (1996). The long-term outcome of a personal network-oriented HIV prevention intervention for injection drug users: the SAFE study. American Journal of Community Psychology.

